# Bifunctional Nitrogen and Cobalt Codoped Hollow Carbon for Electrochemical Syngas Production

**DOI:** 10.1002/advs.201800177

**Published:** 2018-05-07

**Authors:** Xiaokai Song, Hao Zhang, Yuqi Yang, Bin Zhang, Ming Zuo, Xin Cao, Jianhua Sun, Chao Lin, Xiaopeng Li, Zheng Jiang

**Affiliations:** ^1^ School of Chemical & Environmental Engineering Jiangsu University of Technology Changzhou 213001 China; ^2^ Shanghai Synchrotron Radiation Facility Shanghai Institute of Applied Physics Chinese Academy of Sciences Shanghai 201204 China; ^3^ Institute of Coal Chemistry Chinese Academy of Sciences (CAS) Shanxi 030001 China; ^4^ Hefei National Laboratory for Physical Sciences at the Microscale University of Science and Technology of China Hefei Anhui 230026 China; ^5^ CAS Key Laboratory of Low‐Carbon Conversion Science and Engineering Shanghai Advanced Research Institute (SARI) Chinese Academy of Sciences (CAS) Shanghai 201210 China

**Keywords:** bifunctional catalysts, electrochemical CO_2_ reduction reaction (CO_2_RR), hydrogen evolution reaction (HER), metal–organic frameworks, single atom electrocatalysts

## Abstract

Electrochemical conversion of CO_2_ and H_2_O into syngas is an attractive route to utilize green electricity. A competitive system economy demands development of cost‐effective electrocatalyst with dual active sites for CO_2_ reduction reaction (CO_2_RR) and hydrogen evolution reaction (HER). Here, a single atom electrocatalyst derived from a metal–organic framework is proposed, in which Co single atoms and N functional groups function as atomic CO_2_RR and HER active sites, respectively. The synthesis method is based on pyrolysis of ZnO@ZIF (zeolitic imidazolate framework). The excess in situ Zn evaporation effectively prevents Co single atoms (≈3.4 wt%) from aggregation and maintains appropriate Co/N ratio. The as‐prepared electrocatalyst is featured with high graphitic degree of carbon support for rapid electron transport and sponge‐like thin carbon shells with hierarchical pore system for facilitating active site exposure and mass transport. Therefore, the electrocatalyst exhibits a nearly 100% Faradic efficiency and a high formation rate of ≈425 mmol g^−1^ h^−1^ at 1.0 V with the gaseous product ratio (CO/H_2_) approximating ideal 1/2. With the assistance of an extensive material characterization and density functional theory (DFT) calculations, it is identified that Co single atoms are uniformly coordinated in the form of Co–C_2_N_2_ moieties, and act as the major catalytic sites for CO_2_ reduction.

Renewable energy has been developing intensively in the past decade, and is expected to play a dominant role powering human society. However, renewable electricity in many scenarios is timely and spatially decoupled with the customer demand, and its intermittency may jeopardize electric grid stability. Therefore, on‐site converting renewable electricity in the form of chemical energy has attracted enormous interests recently. Electrochemical reduction of CO_2_ and H_2_O into CO and H_2_ is one promising route.^1^, ^2^, ^3^ Mature syngas conversion technologies such as Fischer–Tropsch process can further convert CO and H_2_ (CO/H_2_ = ≈1/2) into value‐added chemicals and fuels.^4^ Combining with geographical distribution of carbon resources (e.g., biomass, coal, tail gas), this technology provides a solution to break the territorial restriction of renewable energy and reduce the carbon emission. Within this context, it is essential to develop affordable electrocatalyst with bifunctional activity for CO_2_ reduction reaction (CO_2_RR) and hydrogen evolution reaction (HER) for technically and economically advanced energy system.

Singe atom electrocatalyst (SAEC) is a new frontier of electrocatalysis.^5^ Metal–organic frameworks (MOFs) have been demonstrated as excellent precursors or carriers for SAECs.^6^, ^7^, ^8^, ^9^, ^10^, ^11^, ^12^, ^13^ In contrast to conventional electrocatalyst in the form of foil, granule, or nanoparticle, SAEC not only brings down material cost in order of magnitude scale, but also displays intriguing physiochemical properties, allowing further manipulation of the activity and selectivity of electrocatalyts.^14^, ^15^, ^16^, ^17^, ^18^, ^19^, ^20^, ^21^, ^22^, ^23^, ^24^, ^25^ Nonetheless, to date, SAECs have been mostly designed as unifunctional catalysts,^14^, ^15^, ^16^, ^17^, ^18^, ^19^, ^20^, ^21^, ^22^, ^23^, ^24^, ^25^ and few SAECs have been experimentally realized possessing bifunctional activity. Chen et al. fabricated atomically dispersed Fe on N‐ and S‐codecorated hierarchical carbon layers which can serve for oxygen evolution reaction and oxygen reduction reaction (OER and ORR), while conventional FeN*_x_*C*_y_* moieties have been known only active for ORR.^26^ Fei et al. fabricated an OER and ORR active NiN_4_C_4_‐embedded graphene sheet, and proposed that OER on NiN_4_C_4_‐embedded graphene sheet proceeded a dual‐site mechanism. The reaction intermediates O* and OH* favorably adsorb at the adjacent C site, while the OOH* is preferentially residing at the Ni atom.^27^ Zheng et al. found single M–N_2_ site supported by C_3_N_4_ (M = Fe, Co, Ni) could serve for both OER and ORR, however, a performance ceiling existed for the M–N_2_ site.^28^ These recent reports suggested that metal single site cannot simultaneously meet the demand for two separate reactions. Creating dual active sites within SAEC could achieve optimized performance for each targeted reaction. Moreover, the synthesis of bifunctional SAEC requires sophisticated control over the catalytic motif, support composition, and microstructure.^26^, ^27^, ^28^, ^29^ It is essential to explore facilitated synthetic approaches that allow rational design of SAECs with tailored activities.

Here, we proposed a novel concept of designing bifunctional Co‐based SAECs derived from MOF by integrating dual active sites. Considering CO_2_RR and HER are two competing reactions, directional generation of CO and H_2_ for syngas production can be achieved on Co single atoms with preferential adsorption of CO_2_,^30^, ^31^ while other nitrogen functional groups (e.g., graphitic and pyridinic N) can serve for HER.^32^ Realizing this, conceptual catalyst demands the formation of high content of Co single atoms with unified coordination chemistry, as well as the presence of HER active nitrogen functional (N—C) groups. Meanwhile, the microstructure of carbonaceous support should be simultaneously engineered in order to allow easy access for CO_2_ molecules and maximize the active site exposure. Conventionally, high temperature pyrolysis of MOF often leads to easy aggregation of metal single atoms into nanoparticles and collapse of porous network, accompanied with the loss of surface area.^33^, ^34^, ^35^ In this work, we reported a facile approach of obtaining a bifunctional Co SAEC supported by nitrogen‐doped 3D hollow carbon structure (denoted as Co‐HNC), and the Co content can reach as high as 3.4 wt% and exclusively coordinated in the form of Co–C_2_N_2_ based on direct pyrolysis of ZnO@ZIF (zeolitic imidazolate framework) heterostructure. The in situ excess Zn evaporation from ZnO template not only effectively prevented aggregation of Co single atoms, but also avoided large N loss during pyrolysis for maintaining appropriate Co/N ratio. Moreover, the as‐prepared Co‐HNC catalyst was featured with a high graphitic degree of carbon support for rapid electron transport, as well as sponge‐like thin carbon shells with hierarchical pore system, which is highly favorable for diffusion of reactants and products. For the application of electrochemical reduction of CO_2_ and H_2_O, the Co‐HNC displayed an excellent Faradic efficiency, a high productivity, and a satisfactory CO/H_2_ ratio. Density functional theory (DFT) calculations together with control experiments were also performed to validate the preferential adsorption of CO_2_ on the Co–C_2_N_2_ moieties.

The preparation of Co‐HNC started with using ZnO nanosphere (ZnO NS) with diameter of ≈160 ± 20 nm as the template. ZnO partially dissolved, and provided Zn source as well as nucleation sites for homogenous growth of bimetallic Zn/Co–ZIF after adding 2‐methylimidazole and Co(NO_3_)_2_ into the ZnO NS suspension (Figure S1, Supporting Information). The coexistence of ZnO and Zn/Co–ZIF in the ZnO@Zn/Co–ZIF nanospheres was confirmed by X‐ray diffraction (XRD) patterns (Figure S2, Supporting Information). The precursor was carbonized under N_2_ atmosphere at 900 °C, in which the ZnO NS was reduced by the in situ formed carbon and subsequently evaporated due to the low boiling point of Zn (907 °C). The excess Zn evaporation was expected to prevent aggregation of Co atoms and maintain porous system in the resultant carbonaceous Co‐HNC. **Figure** **1**a and Figure S3 (Supporting Information) show the scanning electron microscopy (SEM) images of the as‐prepared Co‐HNC, indicating the formation of irregular carbon spheres with a rugged surface and an average diameter of ≈200 nm. Transmission electron microscopy (TEM) (Figure S3, Supporting Information) and scanning transmission electron microscopy (STEM) images (Figure 1b,c) revealed hollow interiors and thin carbon shells with highly porous nature. The shell thickness is around 10–20 nm. Of note, only a small amount of tiny Co particles existed in the Co‐HNC (highlighted in Figure 1b). The electron energy loss (EEL) spectroscopy mapping indicated homogeneous distribution of N, O, and Co throughout the hollow carbon structure (Figure 1d–h). High‐angle annular dark‐field scanning transmission electron microscopy (HAADF‐STEM) with sub‐Ångström resolution revealed the presence of high density of Co single atoms embedded in the carbon shell. The EEL spectrum (Figure 1i) demonstrates that N species are associated with the Co single atoms. In contrast, the carbonaceous product from ZIF‐67 without ZnO core showed “solid” interior, and comprised a large amount of Co nanoparticles (Co NPs) encapsulated nearly in every carbon particle (Figure S4, Supporting Information), which was further confirmed by XRD measurement (Figure S5, Supporting Information). Raman spectroscopy was also conducted. Both Co‐HNC and solid nitrogen‐doped carbon embedded with Co nanoparticle (Co NP‐SNC) shared an identical D‐ to G‐band intensity ratio (*I*_D_/*I*_G_ ≈ 1.0) (Figure S6, Supporting Information), suggesting that in situ reduction and evaporation of ZnO core had no effect on the graphitic degree of carbon.

**Figure 1:**
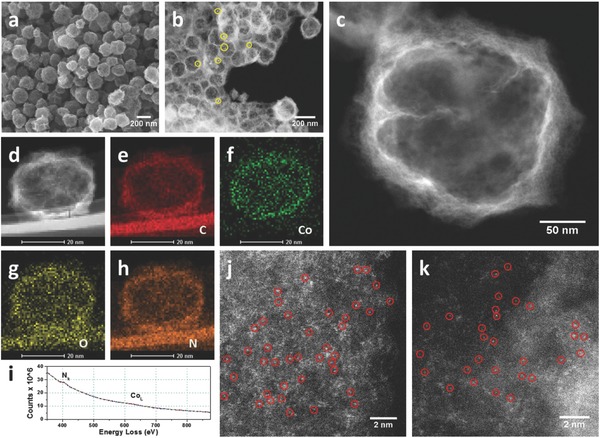
a) SEM image, b) low resolution and c) high resolution STEM image of Co‐HNC. Co NPs are highlighted by yellow circles. d) STEM image of a single hollow carbon sphere and the corresponding EEL spectroscopy element mapping including e) C, f) Co, g) O, and h) N. i) EEL spectrum of Co‐HNC. j,k) HAADF‐STEM images of Co‐HNC at different areas. Part of Co single atoms is marked with red circles.

In order to determine the coordination environment and quantify the ratio of Co single atoms, X‐ray absorption near‐edge spectroscopy (XANES) and extended X‐ray absorption fine structure (EXAFS) were performed and analyzed using the IFEFFIT software.^36^, ^37^ The XANES spectra of Co NP‐SNC (Figure S7, Supporting Information) shared a similar feature as that of Co foil, suggesting that Co species predominantly existed as the metallic phase. A small double peak located at ≈7730 eV indicated that a small amount of Co species existed as oxide phase in the Co NP‐SNC.^38^ In contrast, the Co‐HNC showed different XANES spectra. The white line intensity (at ≈7730 eV) and the associated K‐edge threshold energy of Co‐HNC are significantly higher than those of Co foil and Co NP‐SNC, which evidently proves the dominant ionic nature of Co species in the Co‐HNC. The Fourier transformed (FT) *k* ^3^‐weighted EXAFS spectra of Co‐HNC shows two distinctive peaks. The first peak at 1.45 Å can be ascribed to the CoN*_x_*C*_y_* moieties,^15^, ^20^ and the second one at 2.18 Å belongs to the Co–Co coordination. The Co NP‐SNC displayed a pronounced Co–Co peak without any obvious FT peaks for CoN*_x_*C*_y_*. Based on the EXAFS fitting results (Table S1, Supporting Information), we estimated that ≈84.7% of Co species existed as single atoms in the form of planar Co–C_2_N_2_ in the Co‐HNC,^12^, ^39^ while Co predominantly formed as the metallic nanoparticles in the Co NP‐SNC. This result generally agrees with the STEM observations and highlights the importance of the excess Zn evaporation from ZnO NS.

X‐ray photoelectron spectroscopy (XPS) was carried out to probe the compositional elements and their chemical states. As shown in **Figure** **2**c, the atomic N content of Co‐HNC (3.9 at%) is larger than that of Co NP‐SNC (2.2 at%), suggesting that excess Zn evaporation also prevented N loss. The measured Co atomic contents of Co‐HNC and Co NP‐SNC are 0.31 and 0.22 at%, respectively. Considering XPS has limited detection depth, and Co in the Co NP‐SNC was mostly encapsulated by thick carbon layers, the samples were dissolved by acid and inductively coupled plasma optical emission spectrometer (ICP‐OES) measurement was conducted. The Co contents in Co‐HNC and Co NP‐SNC were determined to be 4.0 wt% (0.8 at%) and 6.9 wt% (1.5 at%), respectively. The deconvoluted high resolution Co 2p spectra of Co‐HNC (Figure 2d) corroborates that Co coordinates with N, as well as the absence of metallic Co, whereas a substantial amount of metallic Co was detected in the Co NP‐SNC. The N 1s spectrum (Figure S8, Supporting Information) was deconvoluted into five types including pyridinic N (398.3 eV), Co–N*_x_* (399.0 eV), pyrrolic N (400.2 eV), graphitic N (401.3 eV), and oxide N (404.2 eV).^35^ The atomic contents of pyridinic N, Co–N*_x_*, pyrrolic N, and graphitic N were 0.550, 0.935, 1.018, 0.908 at% for Co‐HNC, respectively, and 0.639, 0.164, 0.357, and 0.779 at% for Co NP‐SNC, respectively (Figure 2e).

**Figure 2:**
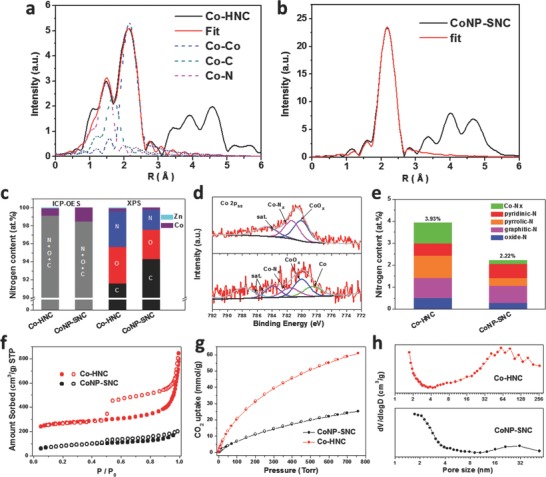
a,b) FT‐EXAFS spectra of a) Co‐HNC, and b) Co NP‐SNC. Red lines represent fitted curves, and dotted lines represent different coordination contributions. c) Element composition of Co‐HNC and Co NP‐SNC measured by ICP‐OES and XPS. d) Co 2p spectra. e) Atomic contents of five different N species in Co‐HNC and Co NP‐SNC. f) N_2_ and g) CO_2_ physisorption isotherms. h) The BJH pore size distribution of Co‐HNC and Co NP‐SNC.

The porous texture of Co‐HNC was analyzed by gas physisorption. The N_2_ sorption isotherm of H‐CoNC exhibited steep adsorption at relatively low pressure and a distinct hysteresis loop at relatively high pressure, indicating the presence of hierarchically porous structure. The Brunauer–Emmett–Teller surface area (*S*_BET_) was determined to be 842 m^2^ g^−1^, which is almost three times higher than that of Co NP‐SNC (280 m^2^ g^−1^). The derived Barrett–Joyner–Halenda (BJH) pore size distribution curve of Co‐HNC from the N_2_ desorption branch evidently shows the hierarchical pores spanning from micro‐ (<2 nm) to macropore (>50 nm) range. The formation of hierarchical pore system in Co‐HNC can be attributed to the excess Zn evaporation from ZnO NS, which effectively prevented loss of surface area and created meso‐ and macropores. For CO_2_RR application, the CO_2_ capture and adsorption capacity of catalysts is of great importance. Figure 2g shows the CO_2_ physisorption isotherms. The Co‐HNC possessed a large CO_2_ capture capacity of 61 mmol g^−1^, which is much larger than that of Co NP‐SNC (25 mmol g^−1^). The CO_2_ physisorption results suggest that the Co‐HNC with hollow and porous nature is permeable for CO_2_ diffusion and following adsorption.

The electrocatalytic reduction of CO_2_ and H_2_O was performed in a gas‐tight full electrochemical cell with catalysts loaded on a porous carbon fiber electrode. **Figure** **3**a shows the cyclic voltammetry (CV) curves recorded in Ar and CO_2_–saturated 0.1 m KHCO_3_ electrolyte. The Co‐HNC exhibited higher current density, more positive onset potential and more pronounced CO_2_RR than Co NP‐SNC. Online gas chromatography (GC) detected two main gaseous products including CO and H_2_. The product formation rate at the cathode as a function of the applied potential was extracted based on controlled potentiostatic electrolysis. As displayed in Figure 3b, the CO + H_2_ formation rate of Co‐HNC increased linearly with the increasing potential and reached as high as ≈425 mmol g^−1^ h^−1^ at 1.0 V versus reversible hydrogen electrode (RHE). The Faradic efficiency (FE) reached nearly 100% when the applied potential was larger than −0.7 V. The CO selectivity also increased with the increasing potential, and maintained ≈35% when the potential was above −0.8 V. The Co‐HNC possessed nice long‐term stability, and negligible degradation of current density was observed after continuous operation for 24 h (Figure 3c). In contrast, Co NP‐SNC displayed an inferior performance, the CO + H_2_ formation rate was nearly half of that for H‐CoNC. Interestingly, we found that the CO selectivity of Co‐HNC generally matched with the Co–N*_x_*/(pyridinic N + graphitic N + Co–N*_x_*) ratio (39.0%). Previous work has shown that the pyridinic and graphitic N atoms have stronger water affinity than other nitrogen species.^40^ Therefore, we speculated that the electrochemical reaction on the Co‐HNC followed a dual‐site mechanism, in which Co–C_2_N_2_ and N functional groups (i.e., pyridinic and graphitic N) function as CO_2_RR and HER sites, respectively. Of note, the function of N functional groups in CO_2_RR is still a matter of debate.^41^, ^42^, ^43^, ^44^ Ongoing work in our group aims to further understand the functionalities of different N—C groups with the presence of metal–N*_x_* centers.

**Figure 3:**
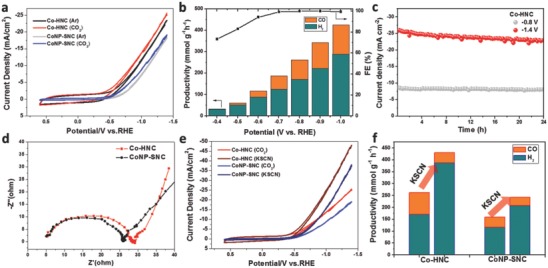
a) CV curves of Co‐HNC and Co NP‐SNC recorded in Ar and CO_2_–saturated 0.1 m KHCO_3_ electrolyte. b) Dependence of productivity (left *Y*‐axis) and FE (right *Y*‐axis) of Co‐HNC on the applied potential. The orange and dark cyan columns represent CO and H_2_, respectively. c) Stability test of Co‐HNC at ‐0.8 V and ‐1.4 V. d) Nyquist plot of Co‐HNC and Co NP‐SNC. e) CV curves of Co‐HNC and Co NP‐SNC recorded in CO_2_‐saturated 0.1 m KHCO_3_ electrolyte with and without KSCN poisoning. f) Formation rate change of Co‐HNC and Co NP‐SNC before and after KSCN poisoning.

To gain insights into the electrocatalytic performance of Co‐HNC, impedance spectroscopy was conducted.^45^, ^46^ Figure 3d shows the Nyquist plot, which indicates a minor difference between Co‐HNC (6.0 Ω) and Co NP‐SNC (5.9 Ω) in series resistance. As revealed by the Raman spectroscopy, both Co‐HNC and Co NP‐SNC had similar graphitic degrees. Therefore, the material conductivity is not a limiting factor and the high conductivity helps realize efficient electron transport in the whole electrode. This is further evidenced by increasing the catalyst mass loading (Figure S9). The productivity of Co‐HNC had a two‐fold increase with a minor change in the CO selectivity when doubling the mass loading (Figure S10). Thus, Co‐HNC should be a promising electrocatalyst for syngas production. The Nyquist plot also provided the evidence of the pore structure, since the low‐frequency inclined slope was associated with mass diffusion behaviors in the electrocatalyst.^47^ Compared to the Co‐HNC, Co NP‐SNC has a lower angle of the slope, suggesting a diffusion limited feature. The hierarchical pore structure of Co‐HNC facilitated the mass transport. In addition, the microstructure features of Co‐HNC including high Co dispersion, large surface area and the sponge‐like thin carbon shell allow maximal exposure of active sites for triple‐phase electrocatalytic reactions. Therefore, Co‐HNC demonstrated a superior gaseous product formation rate over Co NP‐SNC.

In order to confirm Co single atoms and N‐C groups selectively functioning for CO_2_RR and HER, respectively, potassium thiocyanate (KSCN) poisoning experiment was carried out. Figure 3e shows the CV and formation changes upon adding KSCN into electrolyte. Interestingly, the current densities of Co‐HNC and Co NP‐SNC in the CO_2_RR and HER active potential regions significantly increased. Accordingly, the online gas chromatography detected a large increase of gaseous product (Figure 3f), however, the CO selectivity of Co‐HNC dramatically decreased to 9.8%. The CO selectivity for Co NP‐SNC was much smaller. Considering that the SCN^−^ groups can effectively block Co atom, the surrounding two pyridinic N atoms may function as HER sites, leading to the increase of H_2_ production. The pyrrolic N has also been reported as CO_2_RR active sites for CO production,^40^ and it has a large ratio of 41.1% in total active N species (excluding the poisoned Co–N*_x_* and oxide N). Considering the large decrease of CO selectivity, we postulated that Co–C_2_N_2_ should have preferential adsorption of CO_2_ and superior CO_2_RR activity over pyrrolic N.

To further validate that the Co–C_2_N_2_ moieties work mainly as CO_2_RR sites, spin‐polarized DFT was employed to calculate CO_2_ adsorptions via Vienna Ab initio Simulation Package (VASP).^48^, ^49^, ^50^ The projector augmented wave (PAW) pseudopotential was applied for the description of electron–ion interaction,^51^ in junction with generalized gradient approximation (GGA) of Perdew–Burke–Ernzerhof (PBE).^52^ The kinetic cutoff energy was 450 eV for plane‐wave basis and the Monkhorst–Pack scheme generated *k*‐point mesh of 4 × 4 × 1 was employed to describe the Brillouin zone.^53^ All the configurations were relaxed until forces were below 0.05 eV Å^−1^ and convergence criteria for energy were 1 × 10^−5^ eV. A large vacuum space of every two graphene layers in the *z*‐direction was set to 15 Å in case of self‐interactions between neighboring periodic units. **Figure** **4** shows the adsorption of CO_2_ on the Co–C_2_N_2_ moieties embedded in a graphene sheet with several configurations. The binding energy (∆*E*_ad_) was calculated as indicator of the difficulty of CO_2_ binding step, the higher value of ∆*E*_ad_ means more difficulty for CO_2_ adsorption.

(1) $$\Delta \;E_{\mathrm{ad}}\;=\;E_{\mathrm{substrate}\;+\;{\mathrm{CO}}_{2}}\;-\;E_{\mathrm{substrate}}\;-\;E_{{\mathrm{CO}}_{2}}$$

**Figure 4:**
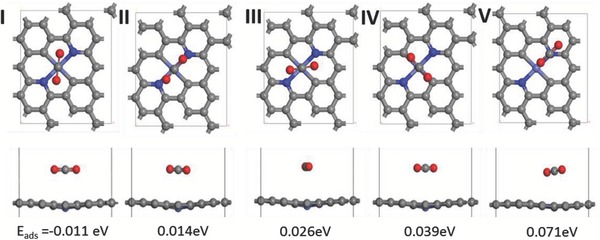
Top‐view and side‐view of CO_2_ molecule adsorbed on Co–C_2_N_2_ sites with different configurations.

Model I has a ∆*E*_ad_ value below zero, indicating spontaneous adsorption, and models II and III require small ∆*E*_ad_ of 0.014 and 0.026 eV, respectively. By contrast, models IV and V demand much higher energy, indicating low probability of existence. Of note, in the models I and II, the C=O length is elongated from original 1.16 to 1.18 Å (Table S2, Supporting Information), indicating preactivation and facilitated disassociation of stable CO_2_ molecule. Above results suggest that the C atom in the linear CO_2_ molecule prefers to bind with the Co atom and two O atoms prefer to be in close proximity with N atoms in the Co–C_2_N_2_. Therefore, the Co–C_2_N_2_ should mainly serve as CO_2_RR sites, rather than act as HER sites based on the theoretical and KSCN poisoning results.

In summary, we have developed a new route to preserve high content of Co single atoms (3.4 wt%) coordinated in the form of Co–C_2_N_2_ and prevent loss of nitrogen through high temperature pyrolysis (900 °C). Therefore, the obtained Co‐HNC can operate as bifunctional catalyst, in which Co–C_2_N_2_ moieties and additional N functional groups (i.e., pryidinic and graphitic N) serve for CO_2_RR and HER, respectively. In addition, its 3D hollow structure and sponge‐like thin shell with hierarchical porous system facilitated the mass transport and maximal exposure of active sites, framing the Co‐HNC as an ideal electrocatalyst for electrochemical reduction of CO_2_ and H_2_O. The gaseous product can reach a formation rate of 425 mmol g^−1^ h^−1^ at 1.0 V versus RHE, and the CO/H_2_ ratio approached around 1/2 in the potential range from −0.7 to −1.0 V. The electrochemical productivity can be improved by simply increasing the applied potential and catalyst loading with a minor change in CO selectivity, which makes the Co‐HNC promising for syngas production. Our work suggests a new route to design bifunctional SAECs, and the insights into the origin of electrocatalytic performance of Co‐HNC are helpful to further design MOF–derived carbonaceous materials for targeted functionalities.

## Experimental Section

*Chemicals and Materials*: 2‐methylimidazole (98%) and Nafion (5 wt%) were purchased from Energy Chemical Co., Ltd. and Sigma‐Aldrich, respectively. Other reagents and solvents were purchased from Sinopharm Co. Ltd., China.

*Preparation of CoSA‐HNC*: ZnO NS with diameter of ≈180 nm was prepared by sonochemical synthesis. Briefly, a 200 mL aqueous solution containing Zn(CH_3_COO)_2_·2H_2_O (4.0 mmol) and triethylamine (TEA, 20.0 mmol) was sonicated vigorously for 30 min at 50 °C. After standing for 12 h at room temperature, the product was centrifugated and washed with water and ethanol, and dried at 60 °C overnight prior to use. The core–shell ZnO@Zn/Co–ZIF precursor was prepared by solvothermal reaction using a mixed solvent of dimethyl formamide (DMF)/H_2_O (3:1 v/v). ZnO NS (1.0 mmol) was first dispersed into 64 mL of solvent by sonication, followed by adding of 2‐methylimidazole (4.8 mmol). After sonication for another 5 min, Co(NO_3_)_2_∙6H_2_O (0.035 mmol) was added into the mixture, then it was heated at 50 °C for 2 h and cooled to room temperature. Finally, the product was centrifugated and washed with fresh ethanol, and dried at 60 °C overnight prior to use. As‐prepared precursor was placed in a tube furnace and heated to 900 °C at a heating rate of 5 °C min^−1^ and kept at this temperature for 2 h under flowing N_2_ (50 cc min^−1^) atmosphere, and cooled naturally to room temperature. The product was then treated with 1.0 m HCl at 80 °C for 3 h to remove the inactive and unstable Zn and/or Co species, and washed thoroughly with water and dried at 60 °C under vacuum overnight for further use.

*Preparation of Co NP‐SNC*: As a comparison, Co NP‐SNC was prepared by a same pyrolysis procedure as described above using ZIF‐67 as precursor. For preparation of ZIF‐67, typically, Co(NO_3_)_2_∙6H_2_O (10 mmol) was dissolved in 200 mL methanol, which was subsequently added into another 200 mL methanol containing 2‐methylimidazole (80 mmol) under vigorous stirring for 1 h. After standing for 12 h, the precipitate was centrifugated and washed with methanol, and dried at 60 °C overnight prior to pyrolysis. The carbonation and acid wash processes were same as that of Co‐HNC.

*Characterization*: The morphology of the samples was characterized by field‐emission SEM (Carl Zeiss Microscopy GmbH Supra 55) and STEM (JEM‐ARM200F). Powder X‐ray diffraction (PXRD) patterns were recorded in a PANalytical diffractometer Model PW3040/60 X'pert PRO using monochromated Cu Kα radiation (40 kV, 40 mA) at a scanning rate of 2° min^−1^. Nitrogen sorption measurement was conducted using a Micromeritics ASAP 2020 system at 77 K. The XPS was performed using the Thermo Scientific K‐Alpha X‐ray photoelelectron spectrometer with Al Kα X‐ray source. Binding energy was calibrated by setting binding energy of C1s peak to 284.8 eV. Raman spectra were acquired with a Horiba Jobin‐Yvon LabRAM HR800 spectrometer using a 514.5 nm laser for excitation. X‐ray absorption fine structure measurements were performed on the beamline BL14W1 in the Shanghai Synchrotron Radiation Facility (SSRF) with the electron storage ring operated at 3.5 GeV, equipped with a double Si (111) crystal monochromator. X‐ray absorption spectroscopy (XAS) data were obtained at fluorescence mode and data were analyzed using the IFEFFIT software package.

*Electrochemical Measurements*: All the electrochemical tests were performed in a gas‐tight electrochemical cell with a typical three electrode configuration. A Pt wire and an Ag/AgCl (Metrohm, 3 m KCl) electrode served as counter and reference electrodes, respectively. To prepare the working electrode, 5 mg of the catalyst was dispersed in water and ethanol mixed solvent (1 mL, v/v = 3:1) with 40 µL Nafion solution (5 wt%). Then, the mixture was ultrasonicated for 60 min to generate a homogeneous ink. Next, the catalyst ink was drop‐casted onto the porous carbon fiber with a 0.5 mg cm^−2^ loading for catalysts. The catalyst mass loading was also corroborated by a balance. The electrolyte was 0.1 m KHCO_3_ aqueous solution. Prior to the tests, the electrolyte was purged with Ar or CO_2_ for at least 20 min. Online gas chromatography (Agilent) was installed for detecting gaseous products including CO and H_2_. CV curves were recorded with a scanning rate of 5 mV s^−1^. The potentials were converted to those versus RHE using *E* (vs RHE) = *E* (vs Ag/AgCl) + 0.1989 V + 0.059 × pH.

*Faradic Efficiency*: The Faradic efficiency for CO and H_2_ production is calculated at a given potential as follows

(2) $$E_{F}\;=\;\frac{J_{\mathrm{CO}}}{J_{\mathrm{total}}}\;=\;\frac{v_{\mathrm{CO}}\;\times \;N\;\times \;F}{J_{\mathrm{total}}}$$

where *J*_CO_ is the partial current density for CO production; *J*_total_ is the total current density; *N* is the number of electron transferred for product formation, which is 2 for CO; ν_CO_ is the production rate of CO (measured by GC); *F* is the Faradic constant, 96485 C mol^−1^; *E*_F_ is the Faradic efficiency for CO production.

## Conflict of Interest

The authors declare no conflict of interest.

## Supporting information

Supplementary
