# Supplementary material for: Bifunctional Nitrogen and Cobalt Codoped Hollow Carbon for Electrochemical Syngas Production
Source: Adv Sci (Weinh). 2018 May 7;5(7):1800177. doi: 10.1002/advs.201800177 (PMC6051375; doi:10.1002/advs.201800177)
Supplement: Supplementary file 1 — Supplementary [file ADVS-5-1800177-s001.pdf]

Copyright WILEY-VCH Verlag GmbH & Co. KGaA, 69469 Weinheim, Germany, 2018.

ADVANCED SCIENCE Open Access

Supporting Information

for *Adv. Sci.,* DOI: 10.1002/advs.201800177

Bifunctional Nitrogen and Cobalt Codoped Hollow Carbon for Electrochemical Syngas Production

Xiaokai Song, Hao Zhang, Yuqi Yang, Bin Zhang, Ming Zuo, Xin Cao, Jianhua Sun, Chao Lin, Xiaopeng Li, and Zheng Jiang**

Supporting information

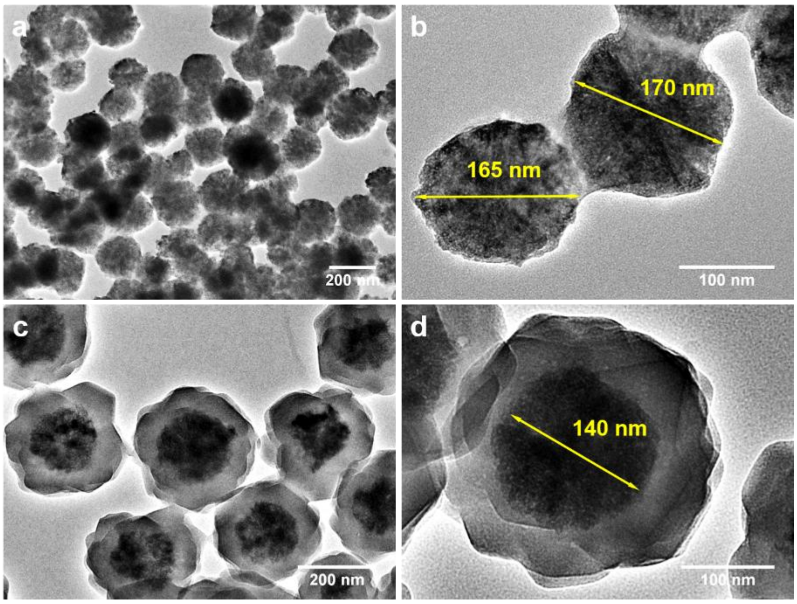

Figure S1. TEM images of (a,b) ZnO nanospheres, and (c,d) ZnO@Zn/Co-ZIF nanospheres.

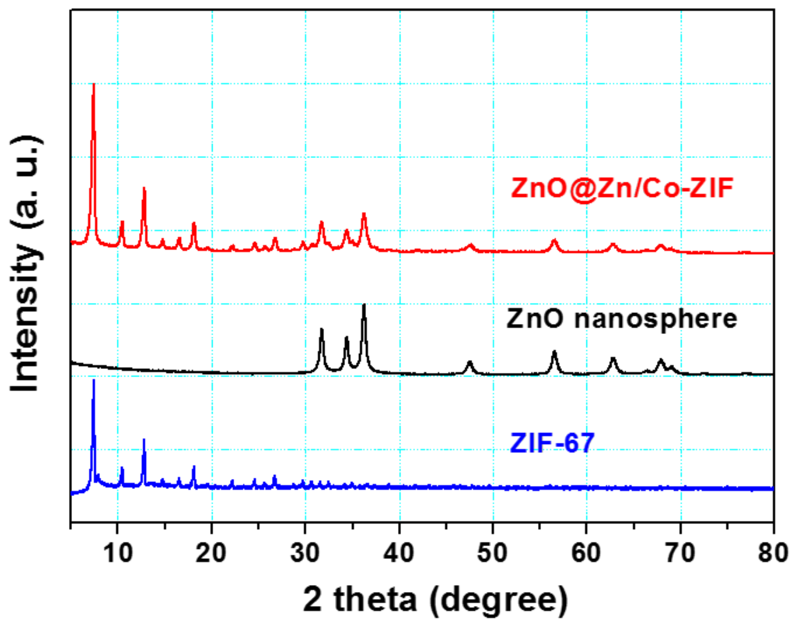

Figure S2. XRD patterns of ZIF-67 (blue), ZnO nanospheres (black) , and ZnO@Zn/Co-ZIF nanospheres (red).

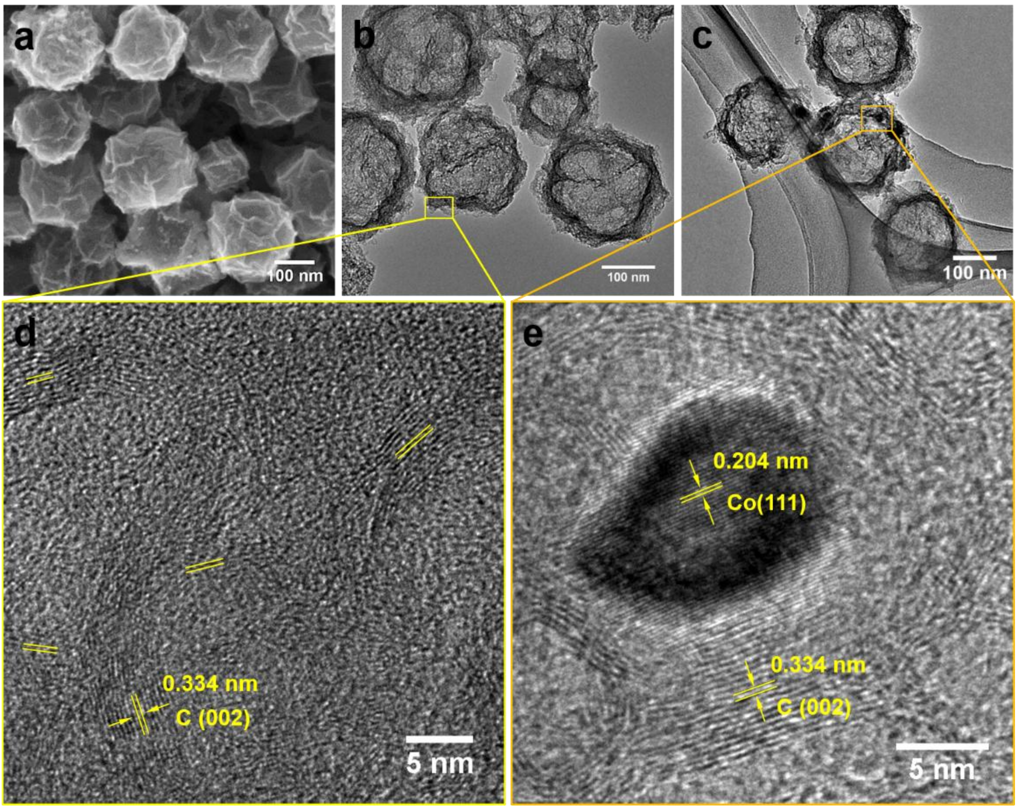

Figure S3. (a) SEM, (b,c) TEM and (d,e) high-resolution TEM images of Co-HNC.

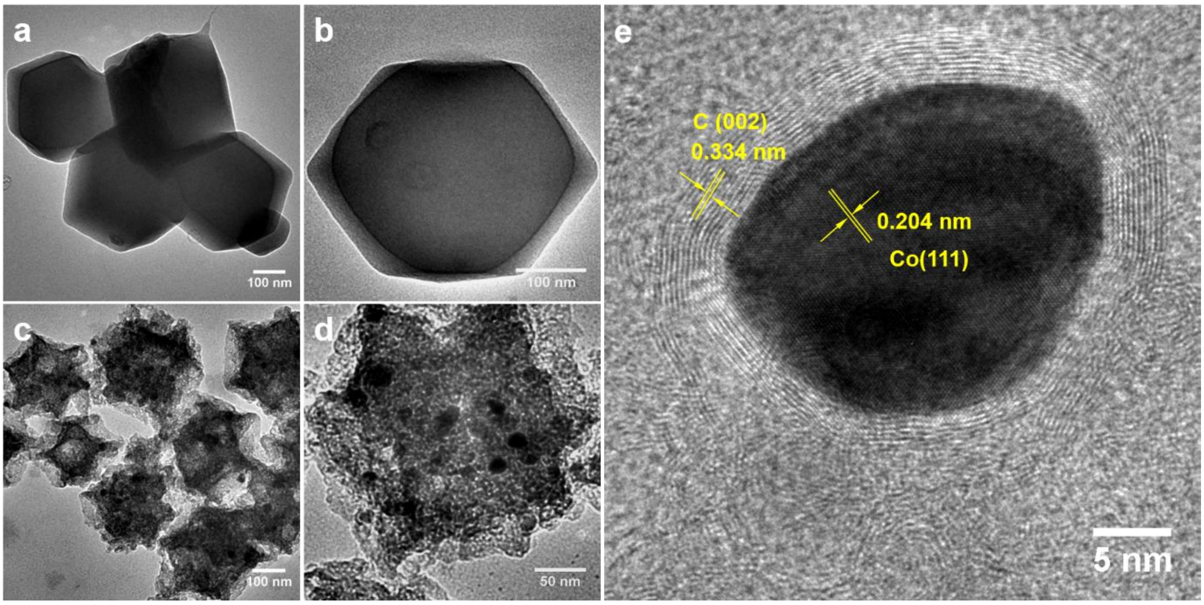

Figure S4. (a,b) TEM images of ZIF-67. (c,d) TEM and (e) high-resolution TEM images CoNP-SNC.

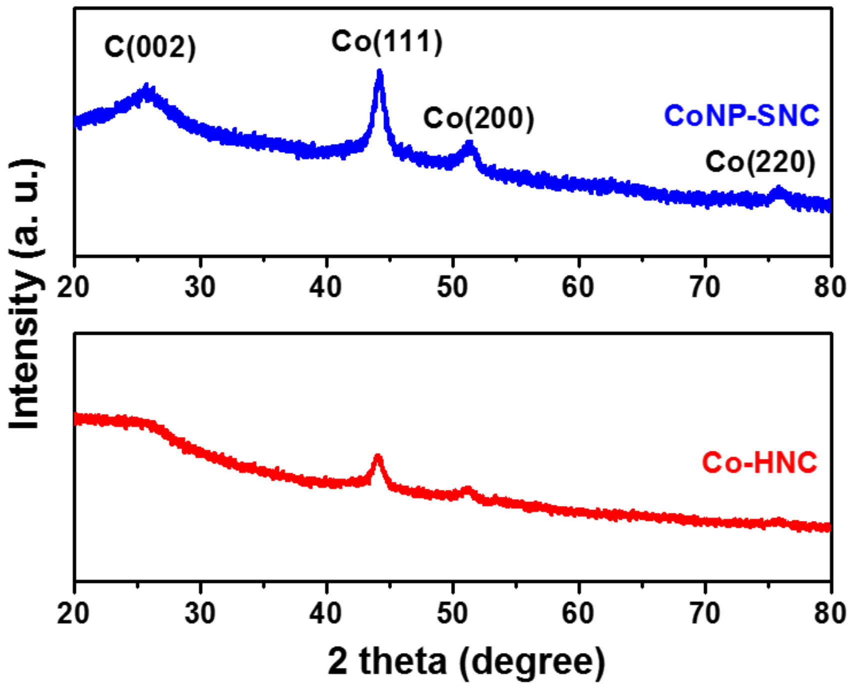

Figure S5. XRD patterns of Co-HNC (red) and CoNP-SNC (blue).

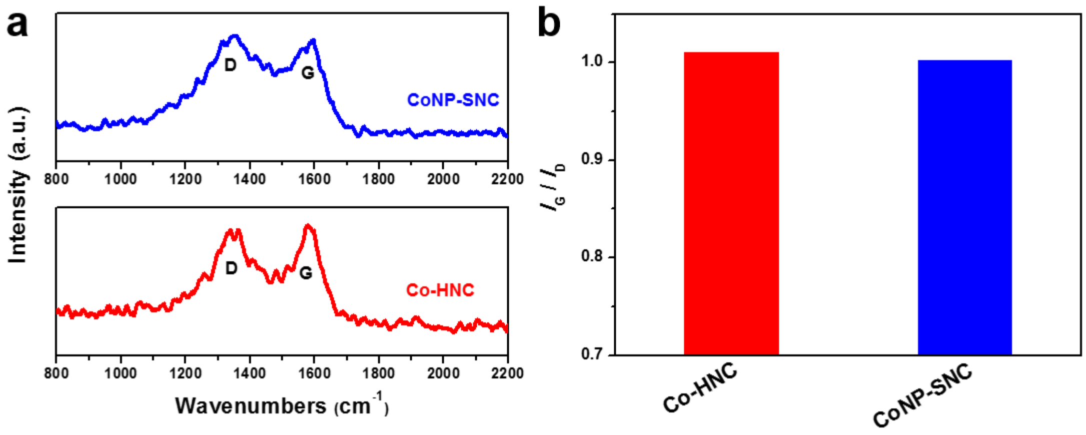

Figure S6. (a) Raman spectra and (b) calculated I_G/I_D values for Co-HNC and CoNP-SNC.

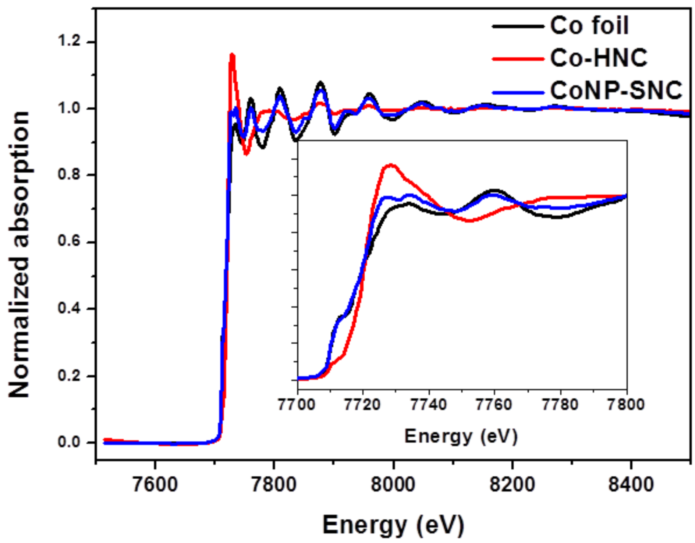

Figure S7. Co K-edge XANES spectra for Co-HNC, CoNP-SNC, and reference Co foil. Insert picture is the enlarged view.

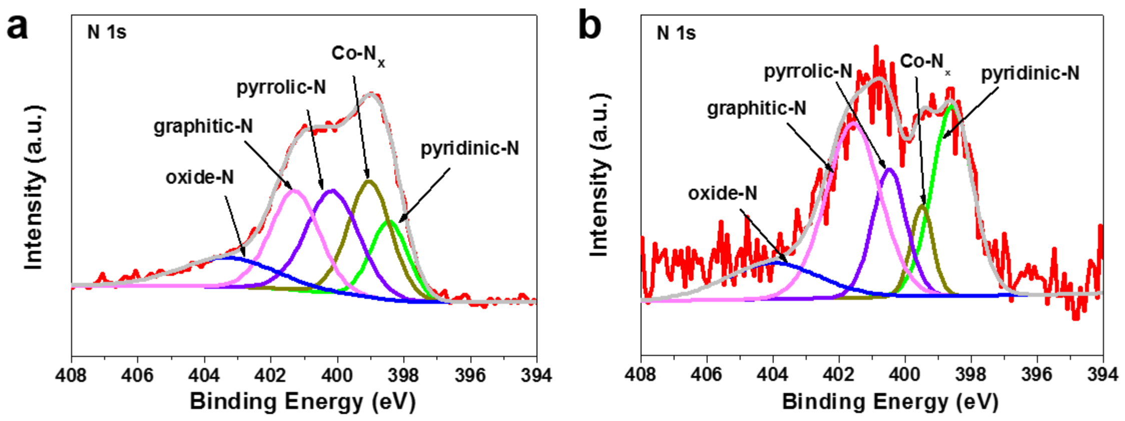

Figure S8. The high-resolution N 1s XPS spectra of (a) Co-HNC, and (b) CoNP-SNC.

Table S1 EXAFS fitting results

Sample	Path	N	R (Å)	ΔE_0 (eV)	σ^2 (10^{-3} Å^2)	R factor
CoNP-SNC	Co-Co	7.4	2.49	7.1	5.3	0.007
Co-HNC	Co-N	2.0	1.92	-3.0	4.8	0.009
	Co-C	2.0	2.10	-3.0	3.0	
	Co-Co	1.3	2.50	-0.3	3.5	

N is the coordination number. R is the distance between absorber and back scatter atoms. ΔE_0 is the inner potential correction to account for the difference in the inner potential between the sample and reference compound. $\Delta\sigma^2$ is the change in the Debye-Waller factor value relative to the Debye-Waller factor of the reference compound.

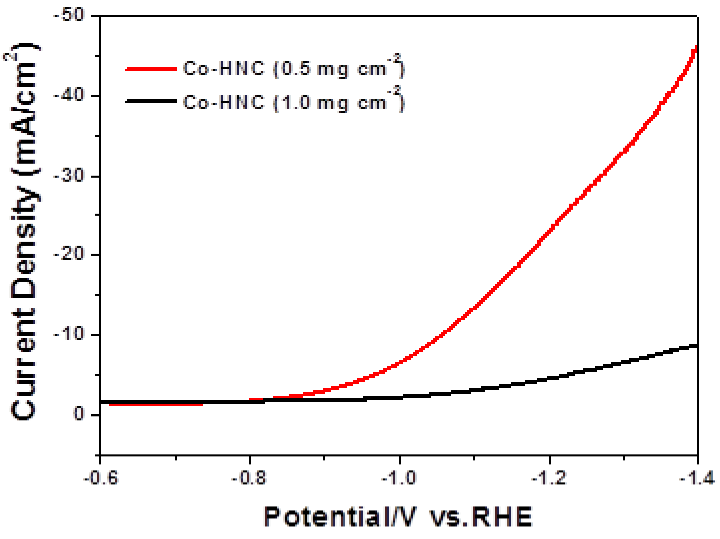

Figure S9. Linear sweep voltammetry of Co-HNC with different catalyst loading mass on the porous carbon fiber electrode.

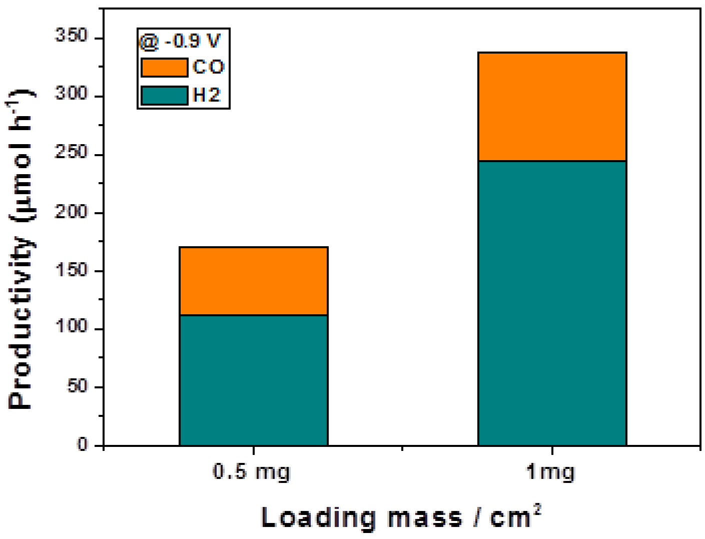

Figure S10. Productivity of Co-HNC with different catalyst loading mass on the porous carbon fiber electrode. The orange and dark cyan columns represent CO and H_2, respectively.

Table S2 C=O bond length (Å) in relaxed structures.

Relaxed structure	C=O bond length (Å)
I	1.1772
II	1.1783
III	1.1754
IV	1.1784
V	1.1765
